# Sex differences in early brain development in a multi-site ASD likelihood cohort

**DOI:** 10.3389/fncom.2026.1868455

**Published:** 2026-08-05

**Authors:** Gabriel Blanco-Gomez, Julie Scorah, Christian O’Reilly, Stefon van Noordt, Virginia Carter Leno, Sara Jane Webb, Mayada Elsabbagh

**Affiliations:** 1 Montreal Neurological Institute-Hospital, Montreal, QC, Canada; 2 Department of Neurology and Neurosurgery, McGill University, Montreal, QC, Canada; 3 Department of Computer Science and Engineering, University of South Carolina, Columbia, SC, United States; 4 Carolina Autism and Neurodevelopment Research Center, University of South Carolina, Columbia, SC, United States; 5 Department of Psychology, Wake Forest University, Winston-Salem, NC, United States; 6 Institute of Psychiatry, Psychology & Neuroscience, King’s College London, London, United Kingdom; 7 Centre for Brain and Cognitive Development, Department of Psychological Sciences, Birkbeck, University of London, London, United Kingdom; 8 Child Health, Behavior and Development, Seattle Children’s Research Institute, Seattle, WA, United States

**Keywords:** autism, EEG, infant development, language development, sex differences

## Abstract

Biological sex is increasingly recognized as a fundamental dimension of brain organization, yet how it influences early neurodevelopment, particularly autism spectrum disorder (ASD), remains understudied. Using data from the International Infant EEG Data Integration Platform (EEG-IP), a multi-site longitudinal cohort of 179 infants (91 males, 88 females) at elevated (ELA) and typical (TLA) likelihood of ASD, we conducted a series of re-analyses of previously published EEG studies to examine whether key EEG metrics differ by biological sex and whether biological sex can moderate the relationship between early brain profiles and language development from 6 to 36 months. Across all EEG metrics, we found that females displayed significantly higher left-hemisphere functional connectivity within speech-related regions at 6 months of age compared to males. Females also demonstrated increased posterior theta consistency during face processing as well as steeper expressive language trajectories from 6 to 36 months. Speech connectivity at 6 months also moderated receptive language growth, but not expressive language growth in females, suggesting sex-specific relationships between brain dynamics and language. These findings show the importance of including biological sex as a primary variable in infant developmental studies and provide support for the study of sex-differentiated biomarkers in prospective ASD studies.

## Introduction

Decades of research strongly support that biological sex can influence brain dynamics and structure (Ingalhalikar et al., 2014). Despite this, only in the last few years has the neuroscience community begun exploring sex as a fundamental aspect of brain organization that goes beyond the influence of sex hormones (Cahill, 2014). In autism spectrum disorder (ASD), a neurodevelopmental condition characterized by deficits in social communication and repetitive or restrictive behaviors (American Psychiatric Association, 2013), the study of sex is particularly important. ASD is diagnosed more often in males than females, with the reported male-to-female ratios at approximately 3:1 (Loomes, Hull and Mandy, 2017). Autistic females are historically more likely to be diagnosed later in life (e.g., adolescence, adulthood) (Carter et al., 2007), exhibit more severe symptoms (Lai et al., 2013) and co-occurring conditions (Dworzynski et al., 2012), suggesting potential phenotypic differences. Recent work suggests that females show social communication deficits that are less obvious as well as repetitive behaviors that are more socially acceptable, resulting in fewer parent concerns (Lai et al., 2015; Hull et al., 2020). Consistent with the sex ratio, research in ASD has historically disproportionately included males, which can undermine developing biomarkers sensitive to ASD in females. In a study of 104 infants (42 males), Bedford et al. (2016) found that previously identified early ASD biomarkers, namely early observational assessments of ASD traits and attentional disengagement were only associated with later symptomatology in boys, underscoring the need to study the effects of sex on emerging ASD traits. These findings highlight a gap in our understanding of the effects of biological sex on brain development and brain-behavior relationships.

Regarding the effects of sex on the brain, studies have shown that autistic females demonstrate more rapid growth in total cerebrum brain volume (Calderoni et al., 2012) and increased functional connectivity (Smith et al., 2019) compared to autistic males. In autistic youth, differences in frontal alpha asymmetry have been correlated with social communication in females (Neuhaus et al., 2023) and autistic male children have shown lower spectral power compared to neurotypical counterparts. In infancy, however, the effects of sex are less well characterized, with some authors reporting no sex effects in EEG spectral power at 3 months of age (Levin et al., 2017).

Using data from the International Infant EEG Data Integration Platform (EEG-IP), a multi-site longitudinal cohort of infants at elevated likelihood of ASD, our team has explored the development of various EEG metrics across infancy. Through a series of studies, we have explored the development of EEG spectral power (Huberty et al., 2021, 2023; Blanco-Gomez et al., 2025a), peak alpha frequency (Carter Leno et al., 2021) and functional connectivity (O’Reilly et al., 2023). However, across this body of work, biological sex has generally been treated as a covariate or addressed in secondary analyses (see Table 1). For example, Huberty et al. (2021) reported that females showed lower spectral power at 6 months, but higher language scores compared to males while O’Reilly et al. (2023) described sex-specific trajectories in functional connectivity and a negative association between functional connectivity and ASD symptom severity at 12 months in females compared to a positive association in males. Finally, using a multivariate clustering approach, our team found a female-enriched cluster defined by high connectivity in speech areas, left-lateralization and overall greater language scores (Blanco-Gomez et al., 2025b). Together, these findings suggest that biological sex might influence early development. The need for this research is exemplified in a recent systematic review of sex differences in ASD, where the authors identified only a handful of comparisons focused on sex differences in brain metrics (Williams et al., 2021). The authors concluded there is a significant gap in understanding how biological sex moderates brain oscillatory activity, which in turn can have cascading effects on symptoms, sex biases in diagnosis and intervention design.

**TABLE 1:** Results from previous studies using EEG-IP data.

Study	EEG Measure	Age (months)	Sample size (n)	Sex tested	Key finding
Huberty et al., 2021	Spectral power	6, 12	397 (208 M/189 F)*	**Yes**	Females showed lower high alpha/beta/gamma at 6 months (*p <* 0.01) and had steeper slopes in alpha and delta (*p <* 0.05).
Carter Leno et al., 2021	Peak alpha frequency	12	127 (66 M/61 F)	Not tested	
van Noordt et al., 2022	Theta phase consistency	6	72 (27M/45 F)	Not tested	
Huberty et al., 2023	Spectral power, Language	6, 12	131 (69 M/ 62 F)	**Yes**	Females had steeper expressive language slope (*p* = 0.006) and higher receptive language intercept at 6 months (*p* = 0.001)
O’Reilly et al., 2023	Functional connectivity	6, 12	182 (94 M/88 F)	**Yes**	There was a marginal sex × age interaction (*p* = 0.055–0.061); females at 12 months showed negative FC–social affect correlation vs. positive in males (although in Seattle only).
Blanco-Gomez et al., 2025a	Lateralization	6, 12	152 (78 M/ 74 F)	Covariate only	
Blanco-Gomez et al., 2025b	Multivariate: Spectral power, Lateralization and Connectivity	6, 12	144 (76 M/68 F)	**Yes**	Females were over-represented in Class A, which had significantly higher speech connectivity (*p* < 0.001) and left lateralization (*p* < 0.05) as well as greater language outcomes.

*EEG-IP is an international platform that combined data from three clinical sites (London, Seattle and Boston). Huberty et al. (2021) used data from all three sites, van Noordt et al. (2022) used data only from the London site while the rest used data from the London and Seattle sites. Bold highlights where sex was test as a primary variable.

In the current study, we address these gaps by examining three questions: First, do EEG measures previously examined in the EEG-IP ASD likelihood cohort (frontal gamma power, functional connectivity across networks [i.e., auditory network, speech network and language network], gamma lateralization, peak alpha frequency and theta phase consistency) differ by sex? Second, where differences in EEG measures are identified, does biological sex moderate the relationship between continuous EEG measures and language development from 6 to 36 months? Finally, given that individual EEG metrics may not capture the full complexity of early brain organization, can a re-analysis of previous multivariate clustering results shed light on how a combination of EEG brain metrics relate to sex-differentiated language trajectories? To address these questions, we conducted a series of re-analyses of studies published using data from the EEG-IP cohort.

## Materials and methods

### Sample

Data were taken from the EEG-IP (van Noordt et al., 2020), a multi-site cohort of infants at elevated likelihood for ASD (ELA) by virtue of having an older sibling with an ASD diagnosis and infants with typical likelihood (TLA). Data were collected at two clinical sites including Birkbeck, University of London (United Kingdom) and the University of Washington-Seattle (United States). A summary of the full cohort and preprocessing procedures can be found in the original publication (van Noordt et al., 2020); below is a summary of the sample used for re-analysis.

This study includes data from 179 infants (91 M, 88 F), including 144 recordings at 6–7 months (6 months hereafter) and 154 recordings at 12–14 months of age (12 months hereafter). A total of 90 infants had elevated likelihood of ASD and 89 were at typical levels of likelihood. Of the infants included in this study, 30 were later diagnosed with ASD based on clinical assessments. ASD diagnoses were ascertained using the Autism Diagnostic Observation Schedule-Generic (ADOS-G). Due to outlier removal, sample sizes vary slightly across EEG metrics (*n* = 140–144 at 6 months and *n* = 154 at 12 months; see Supplementary Table 6 for more information). A full breakdown of participant demographics can be found in the Supplementary Table 1 (see Supplementary Figure 4 for a participant flow diagram). All families provided informed consent prior to participation in accordance with the ethics board of each respective institution.

Language ability in infants was assessed using the Mullen Scales of Early Learning (MSEL), a validated assessment tool used to measure developmental abilities from birth until toddlerhood (Mullen, 1995). Domains from the MSEL include visual reception, fine motor skills, gross motor skills, expressive language and receptive language skills from birth to 68 months of age. For this study, we used only age-equivalent scores from the receptive and expressive language components. Language outcomes were available for a subset of the 179 infants who had EEG data, with the number of available assessments decreasing across time: 144 (80%) had MSEL data at 6 months, 129 (72%) at 12 months, 60 (34%) at 18 months, 100 (56%) at 24 months and 66 (37%) at 36 months. Missingness in MSEL assessments is largely due to site differences in test administration (see Supplementary Table 2). Infants in the London group only received the MSEL at 6, 12, 24, and 36 months, while infants from the Seattle group received the MSEL at 6, 12, 18, and 24 months (only those at ELA received the MSEL at 24 months).

### EEG preprocessing and measures

High-density EEG recordings were collected while participants watched 30–40 s videos using a 128-channel HydroCel Geodesic Sensor Net and the Net Station software (Electrical Geodesics, Eugene, Oregon). Data were preprocessed using the EEG-IP-Lossless pipeline (Desjardins et al., 2021). The present study synthesized analyses across a series of publications using data from the EEG-IP cohort and includes two kinds of EEG measures. 1) measures for which sex-stratified results were reported in the original publication and are shown here and 2) measures where sex was not used as a primary variable of interest and which the original study teams have re-analyzed and incorporated here (see Table 1 for a complete overview of previous analyses).

Four EEG metrics were derived following the same methodology used in Blanco-Gomez et al. (2025b). These include: 1) left-hemisphere functional connectivity between speech-associated regions associated with language ability (Elmer and Kühnis, 2016), 2) inter-hemisphere functional connectivity between auditory cortices, associated with auditory processing (Edgar et al., 2015; Meekings and Scott, 2021), 3) gamma spectral power, associated with excitation-inhibition balance in the brain (Gou et al., 2011) and 4) gamma lateralization which has been shown to represent network efficiency and processing of language information (Gao et al., 2022). All four metrics were available at 6 and 12 months of age.

Peak Alpha Frequency (PAF), which represents the frequency where alpha oscillations reach their maximum amplitude, is associated with cortical maturity and cognitive functioning (Miskovic et al., 2015). This measure was available at 12 months only and data were re-analyzed with sex as a primary variable (for full methodological details see Carter Leno et al., 2021). Moreover, theta phase consistency at 6 months was derived and sex-stratified analyses were done using data from van Noordt et al. (2022). Posterior inter-trial phase consistency during face processing has been linked to selective attention (Zareian et al., 2020) and stimulus encoding for the integration of face information (Kotlewska et al., 2023).

**FIGURE 1:**
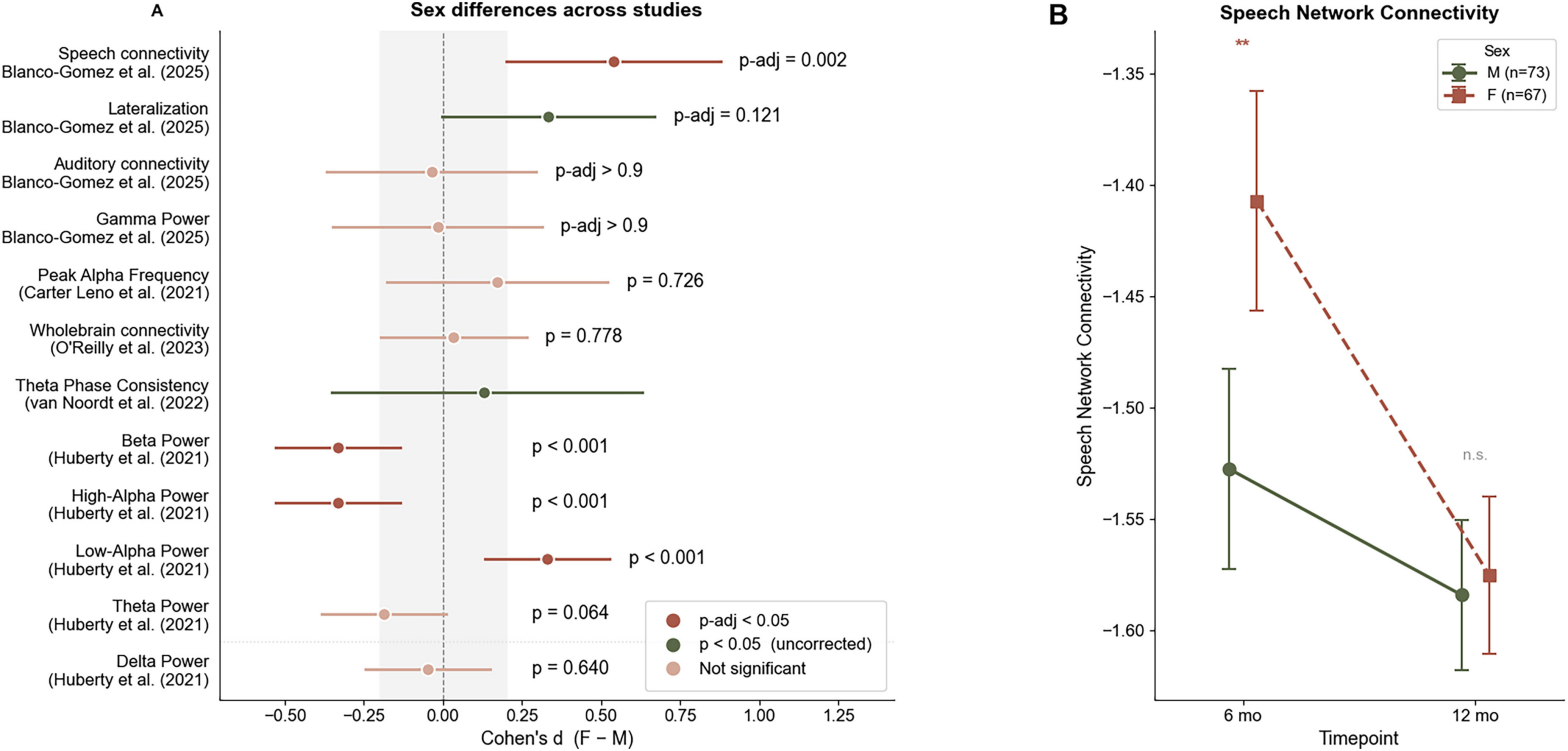
**(A)** Effect sizes (Cohen’s d, female - male) for sex differences in EEG measures across studies. EEG measures analyzed in this study (Speech connectivity, Lateralization, auditory connectivity and Gamma power) display adjusted *p*-values after Holm-Bonferroni corrections, while those taken from other studies represent uncorrected *p*-values (see Supplementary Table 4). Shaded band denotes the small-effect zone (| d| < 0.2). EEG measures presented were derived from 6-month data, except for peak alpha frequency (PAF) values which were assessed at 12 months of age. **(B)** Marginal means ( ± 95% CI) of speech network connectivity at 6 and 12 months of age, stratified by sex. **p < 0.01; n.s., not significant.

Measures of EEG spectral power (broadband, alpha, beta and gamma sub-bands) were examined at 6 months and 12 months in Huberty et al. (2021; *n* = 397) and Huberty et al. (2023; *n* = 131), for which sex-stratified analyses are reported. In addition, functional connectivity was examined across a set of cortical regions in O’Reilly et al. (2023), where the authors found a marginal sex-by-age interaction. Sex effects were included in Figure 1 for contextualization. Readers are referred to these studies for full methodological details.

### EEG subtyping and clustering

Clusters of infants who share similar brain profiles were derived in Blanco-Gomez et al. (2025b) using hierarchical clustering with Ward’s method. These clusters were achieved based on five EEG metrics at 6 months of age including frontal gamma power, gamma lateralization, auditory connectivity, speech connectivity and language connectivity. In this study, three clusters were identified. Cluster A was characterized by high connectivity in the speech network and leftward lateralization; Cluster B was defined by high connectivity in the auditory network, and no lateralization and Cluster C was defined by low connectivity across all networks and no lateralization. Cluster assignments from Blanco-Gomez et al. (2025b) were included without modification in the present study to examine whether early neural profiles at 6 months of age could predict language trajectories from 6 to 36 months as well as later outcomes at 36 months of age. A full demographic breakdown of cluster differences can be found in Supplementary Table 3.

### Statistical analysis

To explore the relationship between biological sex and EEG, linear mixed-effects models were used with subject as a random intercept. Site was used as a covariate and time was centered at 6 months for all analyses. In the full-cohort analyses, where random intercept variance was estimated not to be significantly different from zero (speech connectivity and gamma lateralization), models were refitted as ordinary linear regression. In the ELA subsample analyses, however, the random intercept was estimable for speech connectivity so a mixed-effects model was used. Bonferroni-Holm corrections were also applied for each question family, and estimated marginal means were used to identify significant interactions (see Supplementary Table 5). For exploratory analyses, Benjamini-Hochberg corrections were used. In order to assess language developmental trajectories within clusters, mixed-effects models were fitted both for expressive and receptive language separately, with age, cluster membership, sex, site and non-verbal IQ as fixed effects. Random intercepts per participant were included and interactions between cluster group and age were included to assess differences in language growth curves. Additional analyses were also included to assess outcomes at 36 months in a subset of participants (*n* = 66). Specifically, pairwise sex contrasts by cluster at 36 months were derived from estimated marginal means extracted from the same mixed-effects models used for language trajectories (see Supplementary Table 4; Q3). In all models, males were used as the reference level for sex and London was used as the reference level for site. Full information maximum likelihood was used to deal with missingness in language scores. Finally, neurosubtypes were derived without modification from a prior clustering study (Blanco-Gomez et al., 2025b), wherein groups were derived using hierarchical clustering with Ward’s method. Clustering solutions and optimal number of total clusters/neurosubtypes were determined using the R package *NbClust* (Charrad et al., 2014). All linear mixed-effects models were done using the *lme4* package in R (Bates et al., 2015) while extraction of EEG features was done using *MNE* python package (Gramfort et al., 2013).

## Results

### Sex differences in EEG trajectories

Using a linear mixed-effects model, we examined whether EEG metrics differed by sex at 6 months and 12 months of age. Across all four EEG measures (total of eight tests), only speech network connectivity showed a significant sex difference at 6 months of age (*B* = 0.116, SE = 0.031, *p*-adjusted = 0.002), with females demonstrating higher connectivity than males. Pairwise comparisons confirmed a significant sex difference in speech connectivity at 6 months (*p*-adjusted = 0.0003) with females displaying higher connectivity but no difference at 12 months (*p*-adjusted = 0.788). In terms of trajectories, females showed a steeper decrease from 6 to 12 months, but these differences did not survive multiple corrections (*p*-adjusted = 0.130; see Supplementary Figure 1). No significant sex differences in frontal gamma power, auditory connectivity, or gamma lateralization were observed at 6 months or in trajectories (Supplementary Figure 2 and Supplementary Table 4).

In order to conceptualize findings within the EEG-IP literature, Figure 1A presents effect sizes for sex differences across all measures and studies. A re-analysis of peak alpha frequency at 12 months (Carter Leno et al., 2021) revealed no significant differences between males and females (*B* = –0.028, SE = 0.0791, *p* = 0.726) or interactions between sex and likelihood (*p* = 0.371). Regarding theta phase consistency at 6 months, re-analyses found a sex effect such that females tended to have greater posterior ITC during later stages of visual processing of faces (*p* = 0.017). Sex estimates from O’Reilly et al. (2023) for whole-brain connectivity also indicated no sex differences at the intercept (*p* = 0.778), although they also reported a marginal sex-by-age interaction (*p* = 0.055–0.061). Finally, previous studies using this dataset found significant sex differences in beta power and high-alpha power at 6 months (Huberty et al., 2021), with females showing lower values than males. In addition, females also showed lower low-alpha power compared to males at 6 months.

Moreover, to assess whether ASD likelihood moderated the sex effect on EEG trajectories, we conducted a three-way interaction model including sex, ASD likelihood (TLA vs ELA) and time. Across all four EEG metrics, no interaction survived multiple testing correction (see Supplementary Figure 2). Model fit indices can be found in Supplementary Table 4. In addition, we also conducted an exploratory analysis within the ELA sub-sample (*n* = 90; 47 M, 43 F; 27 ASD, 63 no-ASD) to assess whether sex differences interacted with later ASD diagnosis (*n* = 27). Across all four EEG metrics, we saw significant sex differences in speech connectivity at 6 months (*p*-adjusted = 0.003, Benjamini-Hochberg) with ELA females displaying higher values compared to males at 6 months (Supplementary Figure 1B). Finally, we also found significant sex differences in auditory connectivity at the non-ASD reference level in the ELA infants (*p*-adjusted = 0.0230, Benjamini-Hochberg) with males showing higher connectivity than females, no significant difference was observed among infants who later received an ASD diagnosis. A significant interaction between sex, diagnosis and time was also observed in speech-connectivity (p-adjusted = 0.0257).

### Sex differences in language development

Given that speech connectivity at 6 months was the only metric that differed significantly between sexes, we examined how it moderated language growth from 6 to 36 months, as well as whether this interaction differed by sex. Consistent with previous studies, we found that a large portion of variance in language growth was explained by sex, site, age, non-verbal IQ and connectivity (marginal R^2^ = 0.873 for expressive, 0.877 for receptive), mostly dominated by age.

**FIGURE 2:**
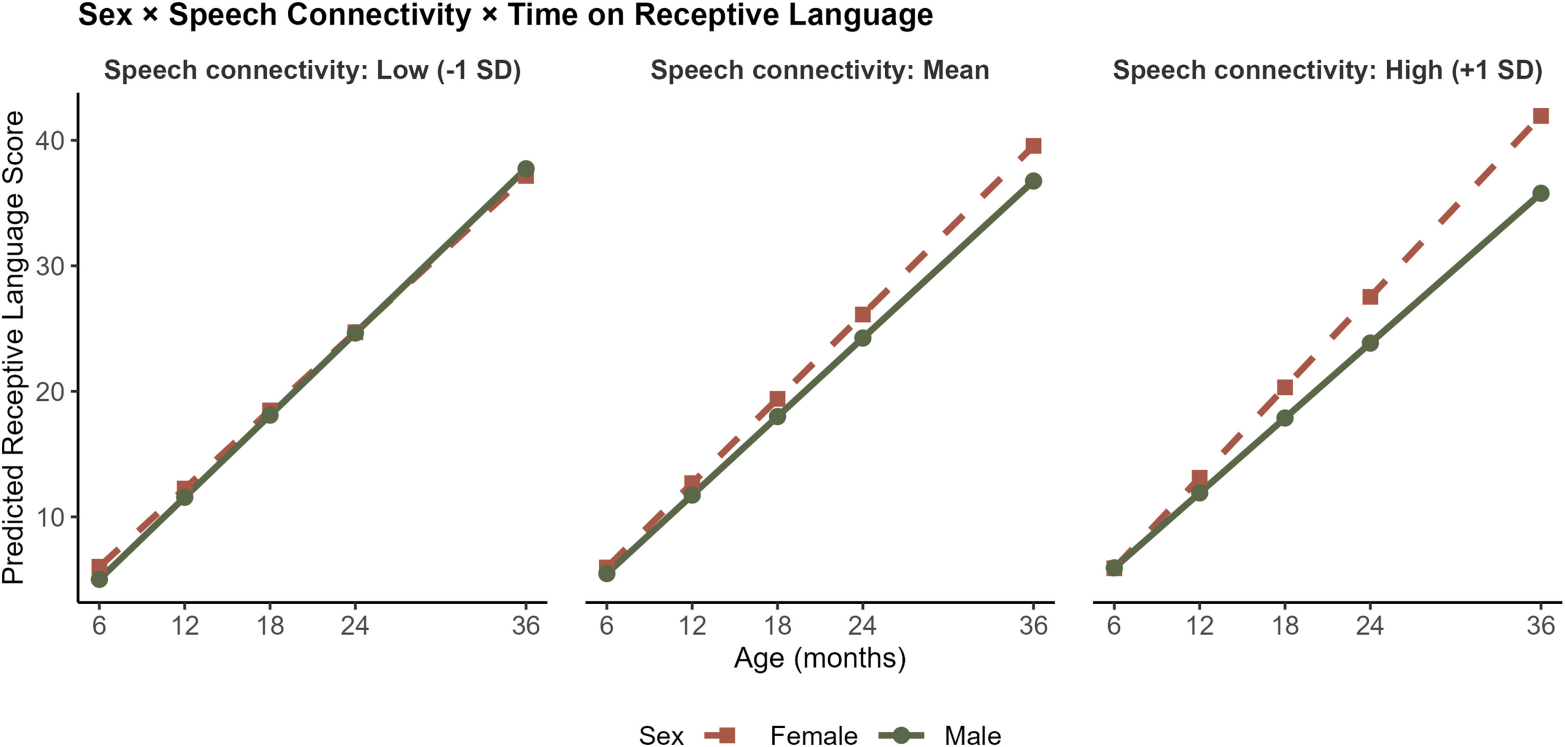
Predicted receptive language growth trajectories by sex and left-hemisphere speech network connectivity at 6 months of age. From left to right, each panel groups infants into three bins based on their speech network connectivity scores: Low (< – 1 SD), Middle (within ± 1 SD), and High (> + 1 SD). Brick red dashed line = Female; olive green solid line = Male. Predictions are derived from linear-mixed effects models with site and non-verbal IQ included as covariates.

In expressive language, we did not find any sex differences at the intercept (*p* = 0.57) and non-verbal IQ was a significant predictor of baseline language ability (*p* < 0.01). We also found a significant interaction between sex and age (*p* < 0.001), wherein females showed greater expressive language growth compared to males. The three-way interaction between age, sex and speech connectivity was not significant (*B* = −0.39, SE = 0.20, p-adjusted = 0.147). Regarding receptive language, we found no sex differences at 6 months (*p* = 0.48), or a significant interaction between sex and age (p-adjusted = 0.147), although non-verbal IQ once again predicted baseline scores (*p* < 0.001). Nevertheless, a significant two-way interaction between speech connectivity at 6 months and age (*p* < 0.05) indicated that higher connectivity was associated with greater receptive language growth. Moreover, a significant three-way interaction between age, speech connectivity and sex (*B* = −0.62, SE = 0.20, p-adjusted < 0.01) suggest that the relationship between connectivity and language growth was moderated by sex (Figure 2). Sex-stratified models also showed that in females, higher speech connectivity at 6 months was associated with faster receptive language growth (*B* = 0.41, SE = 0.11, *p* < 0.001) while in males, this relationship was not significant (*p* = 0.20).

### Sex differences in EEG multivariate profiles

**FIGURE 3:**
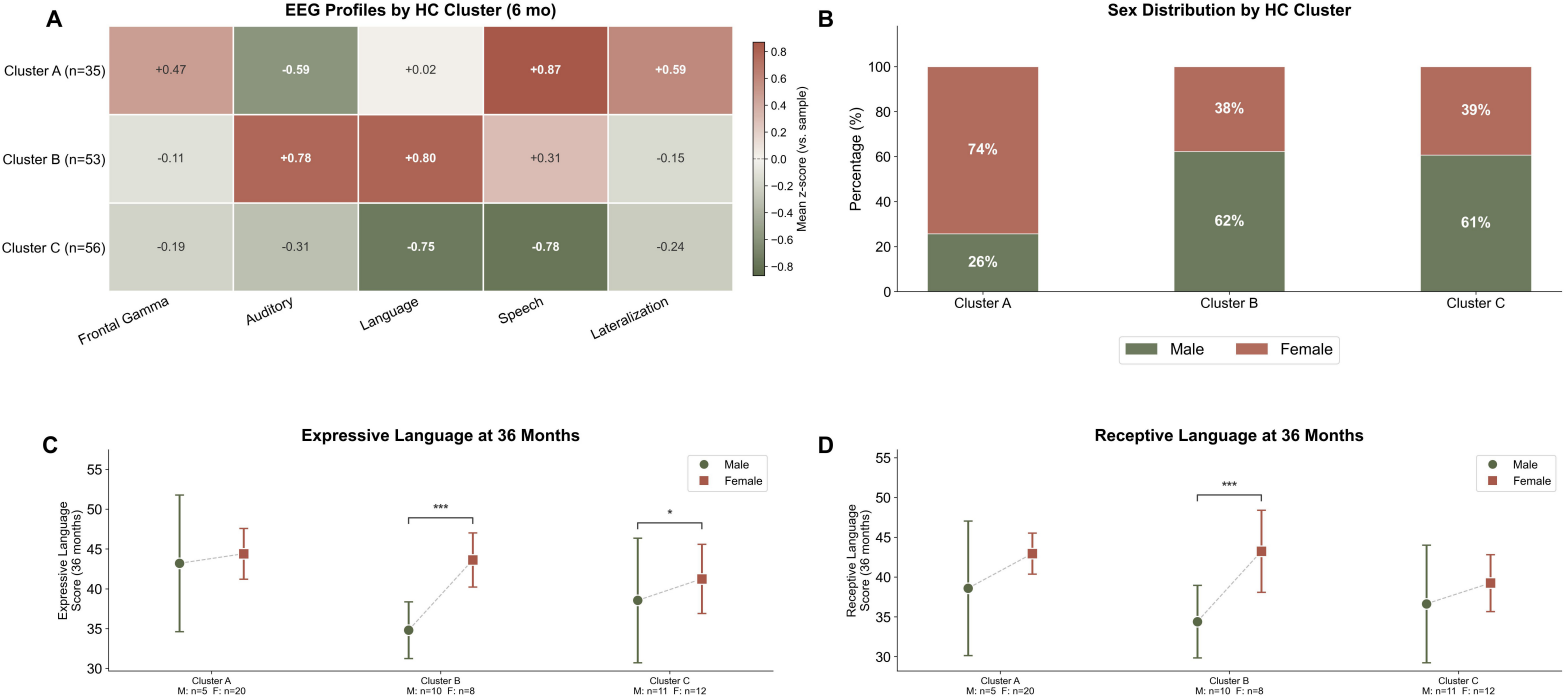
**(A)** Heatmap of mean z-scored EEG features (gamma power, auditory network connectivity, language network connectivity, speech network connectivity, and power lateralization) at 6 months for each cluster. Cell values indicate the standardized deviation from the sample mean. **(B)** Breakdown of sex distributions within each cluster. **(C)** Sex differences in expressive language scores at 36 months of age within clusters. **(D)** Sex differences in receptive language scores at 36 months of age within clusters (***p < 0.001, *p < 0.05).

In order to assess how different EEG metrics might interact with one another to moderate language, we examined the relationship between neurosubtypes (clusters of infants who share similar brain profiles across a series of EEG metrics; Figure 3A), sex (Figure 3B) and language trajectories. Using mixed-effects models, we found a marginal three-way interaction between sex, neurosubtype and time (Expressive: *F*(2, 410) = 3.12, *p* = 0.041, Receptive: *F*(2, 406) = 3.40, *p* = 0.083). However, both expressive and receptive models did not survive corrections (p-adjusted = 0.190 and 0.249, respectively).

Although no differences in language *trajectories* were significant, we conducted exploratory pairwise comparisons to assess whether clusters differed in later language *outcomes* at 36 months of age. Benjamini-Hochberg correction was applied within each language outcome separately (total 3 contrasts per outcome). Analyses showed that in Cluster B, defined by no lateralization and over-connectivity in auditory regions (Figure 3A), there were significant sex differences in receptive language wherein males had lower scores compared to females (M - F = -6.34, SE = 1.60, *n* = 18 (8F), p-adjusted < 0.001; Figure 3D). A similar pattern was found in expressive language (M - F = -6.60, SE = 1.63, n = 18 (8F), p-adjusted < 0.001). In addition, males in Cluster C, defined by low overall connectivity across networks, also showed lower expressive language scores compared to females (M - F = –3.22, SE = 1.45, *n* = 23 (12F), p-adjusted = 0.039; Figure 3C; see Supplementary Figure 3 for full 6–36-month trajectories by cluster). No significant sex differences were observed in Cluster A, a subtype defined by high speech connectivity and strong left-hemisphere lateralization.

## Discussion

The present study re-examined biological sex as a primary variable across a series of EEG analyses in a longitudinal cohort of infants at differential likelihood for ASD. Unlike previous neuroimaging studies that treat biological sex as a nuisance covariate, this study analyzed whether sex differences can influence early brain development and language. Overall, in our full sample, we observed sex differences in functional connectivity within the speech network in the left hemisphere, with females showing higher connectivity at 6 months compared to male infants. In accordance with findings reported in Huberty et al. (2023), females also showed steeper language trajectories compared to males, with higher speech connectivity at 6 months being associated with greater receptive language growth from 6 to 36 months. Exploratory analyses using multivariate approaches also provided preliminary evidence that females show resilience relative to males when displaying EEG profiles associated with lower language scores, though these patterns warrant replication in larger samples. In general, these results reinforce the view that sex differences may influence early brain development with potential downstream consequences for language and broader cognitive trajectories.

## Sex differences in early speech connectivity

Across all the EEG measures analyzed in this study, left-hemisphere connectivity in speech-related areas emerged as the most robust sex difference. At 6 months of age, females across both likelihood groups showed significantly higher speech connectivity compared to male infants. This finding is consistent with a converging body of literature suggesting earlier lateralization and greater myelination in females early in development. While male neonate brains have been shown to have larger cortical thickness (Gilmore et al., 2007) and volume compared to females (Knickmeyer et al., 2014), females undergo accelerated myelination between 3 and 60 months compared to males (Deoni et al., 2012), which may support earlier functional specialization for language. Female infants have also been shown to exhibit more pronounced leftward asymmetries in the prefrontal lobe (Gilmore et al., 2007), serving as a potential mechanism for early language-related lateralization. In our study, we found that speech network connectivity at 6 months was predictive of receptive language growth from 6 to 36 months of age, an effect that was moderated by sex. In females, higher speech connectivity was associated with faster receptive language growth while in males this relationship was not significant. This association between connectivity and language in females could be due to the sex differences in early myelination that facilitate the synchronization between frontal and temporal regions, a process that may not have reached the same degree of maturity in males. Consistent with this, in a study of 53 neonates, Alexopoulos et al. (2022) found that females had greater left-hemisphere activation in response to speech compared to males who showed bilateral activation. Similarly, girls have been repeatedly documented to have advantages in early language development including greater vocabulary growth (Huttenlocher et al., 1991), earlier communicative gestures (Eriksson et al., 2012), and comprehension (Hutchins, 2013); for a review see Rinaldi et al. (2023). The fact that the association between speech connectivity and behavior was only significant for receptive language could suggest that these differences are important earlier on. Developmental studies have shown that comprehension skills emerge earlier than expressive language during the first two years of life (Kuhl, 2010; Rudolph and Leonard, 2016), therefore this dissociation indicates that earlier maturation of left-hemisphere language pathways in females may be relevant for receptive language.

Regarding other EEG measures, our re-analyses did not find any significant differences in 12-month peak alpha frequency, thought to index thalamo-cortical maturation and visual temporal resolution (Freschl et al., 2022). Female infants however, showed increased posterior theta consistency during face processing, suggesting more reliable synchronization of occipito-temporal visual systems during facial identity processing (Kotlewska et al., 2023). As such, this pattern could suggest broadly equivalent global maturation between sexes, but domain-specific differences in face and language processing. In infants, EEG metrics of face processing have been associated with language measures (Glauser et al., 2022; Bosseler et al., 2024), suggesting a potential mechanism for the observed female advantage seen in language. This domain selectivity is consistent with previous studies suggesting that sex effects on the developing brain might be region- rather than network-specific (Deoni et al., 2012; Gilmore et al., 2018). This specificity is also evident in spectral power findings by Huberty et al. (2021) who reported that male infants showed higher beta and high-alpha while females showed significantly higher low-alpha power. Given that spectral power has been associated with attention and language skills (Mundy et al., 2000; Wilkinson et al., 2020), these findings collectively also point to sex-differentiated functional specialization that may emerge prior to the first year of life.

Results from the linear mixed-effects model assessing the interaction between sex, ASD likelihood and time showed no significant differences across all EEG measures. However, exploratory analyses within the ELA subsample suggested that, among infants who did not later receive an ASD diagnosis, females also showed higher speech connectivity and lower auditory connectivity. No significant differences were found among infants later diagnosed with ASD. Siblings of autistic children have been shown to display over-connectivity or under-connectivity to offset risk factors (Kaiser et al., 2010). Given that infants at risk for ASD show enhanced sensitivity to auditory stimuli (Polver et al., 2025), higher auditory connectivity in non-ASD ELA males may reflect up-regulation of an overly sensitive auditory mechanism. Consistent with this interpretation, previous work in this cohort has shown that auditory over-connectivity at 6 months is associated with lower language scores (Blanco-Gomez et al., 2025b). However, whether this reflects a form of early resilience or a distinct developmental pathway remains an open question requiring further investigation. Given the exploratory nature of these analyses, the findings should be interpreted cautiously.

## Neurosubtypes and sex-specific resilience

These patterns converge with the exploratory findings from our neurosubtyping analyses examining whether 6-month EEG profiles predict sex-differentiated language outcomes at 36 months. Results from our previous neurosubtyping study initially revealed three EEG clusters of infants (Blanco-Gomez et al., 2025b). More importantly, these clusters had differing sex compositions. Cluster A, which was characterized by high connectivity in speech regions and leftward lateralization, was approximately 74% female, while Clusters B and C were 38% and 39%, respectively. Interestingly, the brain profile most characteristic of female infants (Cluster A) also displayed the steepest language growth and best language outcomes at age 36 months. In that study, sex was examined as a main covariate of language outcomes, whereas in the present study, we explored how sex moderated language trajectories across neurosubtypes.

In our re-analysis, we found that male infants who belonged to Cluster A did not show the expected sex gap in language scores seen in other groups, thus suggesting that high speech connectivity combined with leftward lateralization at 6 months might be beneficial for language acquisition irrespective of sex. Furthermore, exploratory analyses of language outcomes at 36 months of age showed potential sex differences within Cluster B, a brain profile associated with sub-optimal language performance. Males in this group, characterized by over-connectivity in the auditory network and a lack of lateralization, achieved lower language scores compared to females within the same group. This suggests that females with “sub-optimal language” brain profiles may not be as severely affected as their male counterparts, consistent with theories of female resilience in ASD (commonly referred to as the female protective effect). Evidence for this theory comes primarily from genetic studies, demonstrating that autistic females carry a greater loading of mutations compared to males (Jacquemont et al., 2014). In addition, previous work has shown sex differences in the incidence of intellectual impairments (Dworzynski et al., 2012), behavioral problems (Horiuchi et al., 2014) and EEG patterns (Matlis et al., 2015; Williams et al., 2021). Results from our study offer preliminary evidence to this literature, suggesting that even in the presence of EEG patterns that are not usually optimal for early language acquisition (e.g., auditory over-connectivity and a lack of lateralization), female infants may still follow typical language trajectories or at least achieve typical scores as measured through standardized testing. Nevertheless, given our sample size and cohort profile, larger confirmatory samples are needed to further understand these effects. Given that females are less likely to be diagnosed early (Mandic-Maravic et al., 2015) future studies should include more representative samples of autistic girls and women and acknowledge how these differences can affect the interpretation of clinical findings.

### Limitations

First, we should acknowledge the difficulties of collecting and analyzing EEG in infants. Potential confounds in this age group include poor fit of EEG caps, differences in skull connectivity due to heterogeneity in developmental stages, failure to locate reference electrodes, differences in canonical EEG rhythms (e.g., lower boundaries for alpha power) among other factors (Noreika et al., 2020). Second, known sex differences in ASD diagnosis rates could also affect the results. Females are more likely to be diagnosed later in childhood and adulthood (Carter et al., 2007) while in this study, diagnostic outcome was only assessed at age 3. Consequently, future longitudinal follow-up may reveal a higher proportion of females receiving an ASD diagnosis. Moreover, Cluster A was primarily female (74%) and was defined mainly by differences in speech connectivity. This suggests that the cluster itself may carry sex-related variance and as such, sex x cluster findings should be interpreted with caution. Finally, only one site had language data at 36 months of age (*n* = 66), thus the neurosubtyping results should be interpreted with caution.

## Conclusion

The current study provides empirical evidence for sex differences in early brain development across a series of EEG metrics from the EEG-IP longitudinal cohort. Overall, we found differences in the functional connectivity of language-related regions in the left hemisphere, with females displaying significantly higher connectivity at 6 months compared to males and increased theta activity during face processing. We also found that biological sex moderated the relationship between early speech connectivity and receptive language development, with female infants showing a stronger association. Moreover, exploratory neurosubtyping analyses indicate possible resilience in females, who tended to achieve comparatively higher language outcomes than males with EEG profiles generally associated with lower language performance, though these patterns warrant replication in larger samples. These results highlight the need to include biological sex as an important variable of interest in infant neuroimaging research and make the case for sex-differentiated biomarkers in the study of ASD.

## Data Availability

The raw data supporting the conclusions of this article will be made available by the authors upon request.
