# Supplementary material for: Sex differences in early brain development in a multi-site ASD likelihood cohort
Source: Front Comput Neurosci. 2026 Aug 5;20:1868455. doi: 10.3389/fncom.2026.1868455 (PMC13486256; doi:10.3389/fncom.2026.1868455)
Supplement: Supplementary file 1 [file Data_Sheet_1.pdf]

Supplementary material

Supplementary Table 1

Participant demographics

Age	Group	Outcome	N (females)	Total
6-7 months	ELA	ASD	19 (8)	69
		No ASD	50 (23)	
	TLA	ASD	3 (1)	75
		No ASD	72 (36)	
	Combined	ASD	22 (9)	144[++]
		No ASD	122 (59)	
12-14 months	ELA	ASD	24 (11)	78
		No ASD	54 (26)	
	TLA	ASD	2 (1)	76
		No ASD	74 (41)	
	Combined	ASD	26 (12)	154[++]
		No ASD	128 (66)	

ASD: Autism Spectrum Disorder; ELA: Elevated likelihood for ASD; TLA: Typical Likelihood
[++] This table includes the full sample of participants; minor variation in sample size across EEG metrics reflects outlier removal or missing data for each variable (6–7 months: n = 140–144 depending on the EEG measure; see Supplementary Table 6). At 6 months of age, 4 outliers were removed for all speech and auditory connectivity.

Supplementary Table 2

Breakdown of language variables out of 179 infants involved in the study

Variable	N with data	N missing (%)	Female (%)	Male (%)	ASD diagnosis (%)	ASD likelihood (%)	Mean Expressive ± SD	Mean Receptive ± SD
**MSEL 6 months**	144	35 (19.6%)	68 (47.2%)	76 (52.8%)	22 (15.3%)	69 (47.9%)	6.14 ± 1.89	5.93 ± 1.74
**MSEL 12 months**	129	50 (27.9%)	63 (48.8%)	66 (51.2%)	21 (16.3%)	64 (49.6%)	12.39 ± 2.76	12.29 ± 2.93
**MSEL 18 months**	60	119 (66.5%)	22 (36.7%)	38 (63.3%)	10 (16.7%)	32 (53.3%)	17.27 ± 4.16	18.87 ± 4.61
**MSEL 24 months**	100	79 (44.1%)	51 (51.0%)	49 (49.0%)	17 (17.0%)	66 (66.0%)	24.73 ± 5.84	26.39 ± 5.66
**MSEL 36 months**	66	113 (63.1%)	40 (60.6%)	26 (39.4%)	11 (16.7%)	34 (51.5%)	41.21 ± 8.79	39.64 ± 8.42

Supplementary Table 3

Demographic characteristics of infant neural subgroups identified through hierarchical clustering

Variable	Statistics	Chi-square	p-value	Cramer's V	Class a	Class b	Class c
**ASD Diagnosis**							
ASD	n (%)	0.05	0.974	0.02	5 (15.2%)	8 (16.0%)	9 (17.0%)
no-ASD	n (%)				28 (84.8%)	42 (84.0%)	44 (83.0%)
**ASD Likelihood**							
ELA	n (%)	0.77	0.682	0.073	19 (54.3%)	24 (45.3%)	26 (46.4%)
TLA	n (%)				16 (45.7%)	29 (54.7%)	30 (53.6%)
**Sex**							
F	n (%)	13.61	0.001	0.307	26 (74.3%)	20 (37.7%)	22 (39.3%)
M	n (%)				9 (25.7%)	33 (62.3%)	34 (60.7%)
**Site**							
London	n (%)	11.82	0.003	0.287	25 (71.4%)	19 (35.8%)	23 (41.1%)
Seattle	n (%)				10 (28.6%)	34 (64.2%)	33 (58.9%)

Supplementary Table 4

Results from linear models

EEG Variable	Comparison	Test type	Model	p (raw)	p (Holm)	p (BH)	R^2	Sig.
**Q1.1: Sex differences in EEG Development**								
Frontal Gamma Power	*Sex at 6 mo*	Intercept	lmer	0.9585	1.0000		0.3796	ns
Auditory Network	*Sex at 6 mo*	Intercept	lmer	0.9372	1.0000		0.0945	ns
Speech Network	*Sex at 6 mo*	Intercept	lm*	**0.0002**	0.0020		0.1458	**
Power Lateralization	*Sex at 6 mo*	Intercept	lm*	0.0172	0.1207		0.0441	ns
Frontal Gamma Power	Sex × Time	Interaction	lmer	0.8021	1.0000		0.3796	ns
Auditory Network	Sex × Time	Interaction	lmer	0.6547	1.0000		0.0945	ns
Speech Network	Sex × Time	Interaction	lm*	0.0216	0.1296		0.1458	ns
Power Lateralization	Sex × Time	Interaction	lm*	0.4212	1.0000		0.0441	ns
**Q1.2: Sex differences and ASD likelihood interactions**								
Frontal Gamma Power	*Sex at 6 mo (TLA ref)*	Intercept	lmer	0.9636	1.0000		0.3817	ns
Auditory Network	*Sex at 6 mo (TLA ref)*	Intercept	lmer	0.0230	0.1842		0.1385	ns
Speech Network	*Sex at 6 mo (TLA ref)*	Intercept	lm*	0.0892	0.5353		0.1642	ns
Power Lateralization	*Sex at 6 mo (TLA ref)*	Intercept	lm*	0.0692	0.4842		0.0509	ns
Frontal Gamma Power	Sex × Group × Time	Interaction	lmer	0.5448	1.0000		0.3817	ns

Auditory Network	Sex × Group × Time	Interaction	lmer	0.2908	1.0000		0.1385	ns
Speech Network	Sex × Group × Time	Interaction	lm*	0.2536	1.0000		0.1642	ns
Power Lateralization	Sex × Group × Time	Interaction	lm*	0.2839	1.0000		0.0509	ns
**Q1.3: Sex differences and ASD diagnosis interactions**								
Frontal Gamma Power	*Sex at 6 mo (no-ASD ref)*	Intercept	lmer	0.1604		0.2139	0.4768	ns
Auditory Network	*Sex at 6 mo (no-ASD ref)*	Intercept	lmer	**0.0058**		0.0230	0.1495	*
Speech Network	*Sex at 6 mo (no-ASD ref)*	Intercept	lmer	**0.0004**		0.0032	0.2723	**
Power Lateralization	*Sex at 6 mo (no-ASD ref)*	Intercept	lm*	**0.0171**		0.0342	0.1843	.
Frontal Gamma Power	Sex × Dx × Time	Interaction	lmer	0.2436		0.2784	0.4768	ns
Auditory Network	Sex × Dx × Time	Interaction	lmer	0.1093		0.1750	0.1495	ns
Speech Network	Sex × Dx × Time	Interaction	lmer	**0.0096**		0.0257	0.2723	*
Power Lateralization	Sex × Dx × Time	Interaction	lm*	0.4487		0.4487	0.1843	ns
**Q2.1: Speech Connectivity × Sex × Time on language growth**								
Expressive	Sex x Time	Interaction	lmer	1.78e-06	< 0.001		0.873	***
Expressive	Sex x Time x Speech	Interaction	lmer	0.0575	0.147		0.873	ns

Receptive	Sex x Time	Interaction	lmer	0.0541	0.147		0.877	ns
Receptive	Sex x Time x Speech	Interaction	lmer	0.002	0.007		0.877	**
Receptive	Interaction 36 months	Interaction	lmer	0.0489	0.147		0.152	ns
**Q3: Sex differences in language trajectories by Neurosubtype**								
Expressive	Sex × Cluster	Interaction	lmer	0.725	0.972		0.872	ns
Expressive	Sex × Time	Interaction	lmer	0.0064	0.038		0.872	*
Expressive	Sex x Time x Cluster	Interaction	lmer	0.041	0.190		0.872	ns
Receptive	Sex × Cluster	Interaction	lmer	0.486	0.972		0.873	ns
Receptive	Sex × Time	Interaction	lmer	0.038	0.190		0.873	ns
Receptive	Sex x Time x Cluster	Interaction	lmer	0.083	0.249		0.873	ns
**Q3: 36-month pairwise comparisons**								
Expressive	M − F	Post-hoc (Cluster A)		0.986		0.986		ns
Expressive	M − F	Post-hoc (Cluster B)		0.0000592		<0.001		***
Expressive	M − F	Post-hoc (Cluster C)		0.0251		0.039		*
Receptive	M − F	Post-hoc (Cluster A)		0.538		0.640		ns
Receptive	M − F	Post-hoc (Cluster B)		0.0000821		<0.001		***

Receptive	M − F	Post-hoc (Cluster C)		0.640		0.640		ns

Note: Holm correction applied within each question family. Q1: 4 sex-at-6mo + 4 sex×time. Q1.2: 4 sex×ASD-likelihood + 4 sex×ASD-likelihood×time. Q1.3: 4 sex×ASD-diagnosis + 4 sex×ASD-diagnosis×time. Q2.1: Holm correction applied across the 5 tests. Q3: Holm correction applied across the 6 tests shown (expressive and receptive sex×cluster and sex×time×cluster interactions). p (BH) shown for Q1.3 and 36-month pairwise comparisons(exploratory). lm* = singular lmer replaced with ordinary linear model (random intercept variance = 0). R^2 = marginal R^2 for lmer models; ordinary R^2 for lm* models. Pairwise contrasts were estimated marginal means derived from the corresponding mixed-effects models. Q1.2 and Q1.3 intercept tests reflect sex difference in reference group (TLA and no-ASD respectively).

Supplementary Table 5

Statistical models

Site coding: London = reference category in all models. Missing data: FIML via lmer for all mixed models; complete-case at relevant timepoint for OLS models. Time centred at 6 months in all longitudinal models. Male = reference for sex in Q1–Q1.3; Female = reference in Q2.1.

EEG Measure	Source	Operational Definition	Analytic N (6mo / 12mo)	Statistical Model	Correction Family
**Frontal Gamma Power**	New	Source-estimated (eLORETA) spectral power in the gamma band (30–80 Hz) averaged across frontal electrode clusters, computed from resting EEG during passive video viewing.	144 / 154 (76M/68F at 6mo)	LMER: Y ~ time_c × sex + site + IQ + (1|subject) Site dummy-coded (London = reference). Time centred at 6 months.	Q1, Q1.2, Q1.3
**Gamma Lateralization**	New	Frontal hemispheric asymmetry index: LI = (left − right gamma power) / (left + right gamma power), where left and right refer to homologous frontal electrode clusters.	144 / 154 (76M/68F at 6mo)	OLS regression (random-intercept variance ≈ 0, refitted to OLS): Y ~ time_c × sex + site + IQ Site dummy-coded (London = reference).	Q1, Q1.2, Q1.3
**Speech Connectivity**	New	Corrected imaginary part of the phase-locking value (CiPLV) between left-hemisphere frontal and temporal regions comprising the speech production/sensorimotor network, derived via eLORETA source reconstruction.	140 at 6mo (73 TLA / 67 ELA; TLA: 37M/36F ELA: 36M/31F)	OLS regression (random-intercept variance ≈ 0, refitted): Y ~ time_c × sex + site + IQ (Q1); language ~ time × speech_con_c × sex + site + IQ + (1|subject) (Q2.1). Speech connectivity mean-centred across Q2 sample.	Q1, Q2.1
**Auditory Connectivity**	New	Inter-hemispheric CiPLV between bilateral auditory cortex regions (superior temporal gyrus), reflecting coherence within the auditory/speech-perception network.	140 / 154 (73M/67F at 6mo)	LMER: Y ~ time_c × sex + site + IQ + (1|subject) Site dummy-coded (London = reference).	Q1, Q1.2, Q1.3
**Peak Alpha Frequency (PAF)**	Re-analysed from *Carter Leno et al. (2021)*	Frequency (Hz) at which the posterior alpha power spectrum reaches its maximum amplitude, extracted from posterior electrode clusters at 12 months; reflects cortical maturity and cognitive functioning.	127 at 12mo (66M / 61F)	OLS (single timepoint): PAF ~ sex + site + age-at-visit + ASD-likelihood × sex. Site dummy-coded (London = reference). Model specification unchanged from original publication.	Contextualisation only (no Holm correction applied)
**Theta Phase Consistency**	Re-analysed from *van Noordt et al. (2022*	Mean inter-trial phase consistency (ITC) in the theta band (3–6 Hz) at bilateral posterior electrodes, time-locked to face-stimulus onset; extracted for early (0–200 ms) and late (200–500 ms) post-stimulus windows.	72 at 6mo (27M / 45F)	Sex re-analysis conducted by original study team using original model structure. Females showed greater late-stage (200–500 ms) posterior theta ITC than males ($p = .017$). Model specification unchanged.	Contextualisation only
**EEG Neurosubtypes (Hierarchical Clusters)**	Re-analysed from *Blanco-Gomez,*	Three neurosubtypes derived via hierarchical clustering (Ward's method; optimal k by NbClust majority vote across 30 indices) on five 6-month EEG features	144 at 6mo (cluster assignment)	LMER: language ~ time × sex × cluster + site + IQ + (1|subject). Cluster (A/B/C) as three-level factor. Fitted separately for	Q3

	Wright et al. (2025	(frontal gamma power, gamma lateralization, auditory connectivity, speech connectivity, language connectivity). Cluster assignments used without modification.		expressive and receptive language. Site dummy-coded (London = reference). Model specification unchanged from original publication. Estimated marginal means from these models were additionally used to extract pairwise sex contrasts by cluster at 36 months (n = 66)	
**Spectral Power (broadband + band-specific)**	Context only from *Huberty et al. (2023)*	Broadband and frequency-specific (alpha, beta, gamma) EEG spectral power across scalp regions computed from resting EEG at 6 and 12 months.	131 / 131 (69M / 62F)	Not re-analysed. Sex-stratified estimates from original publication presented for contextualisation only.	Contextualisation only (no Holm correction)
**Functional Connectivity (12 months)**	Context only *O'Reilly et al. (2023)*	EEG functional connectivity across predefined cortical region pairs at 12 months; marginal sex-by-age interaction reported in original publication.	182 at 12mo (94M / 88F)	Not re-analysed. Sex-stratified estimates from original publication presented for contextualisation only.	Contextualisation only (no Holm correction)

Supplementary Table 6

Analysis stage	Subgroup	N	Male	Female
Enrolled	Total	179	91	88
ASD likelihood	TLA	89	44	45
	ELA	90	47	43
EEG recordings	At 6 months	144	76	68
	At 12 months	154	75	79
	Both timepoints	119	60	59
ASD outcome (ELA only)	no-ASD	63	32	31
	ASD	27	15	12
Q1/Q1.2 analysis sample	All	179	91	88
Q1.3 Analysis sample	ELA with outcome	90	—	—
Q2.1 analysis sample	Has speech-con 6mo	140	—	—

	TLA	73	37	36
	ELA	67	36	31
Q3 analysis sample	Has cluster assignments	144	76	68
Language follow-up (36 mo)	Expressive	66	26	40
	Receptive	66	26	40

Model formulas

All models were computed in R (https://github.com/gabrielblancogomez/eegip_sex_diff). Time was centred at 6 months, so coefficients at the intercept correspond to 6-month values. Male was the reference level for sex in Q1–Q1.3; female was the reference level in Q2.1.

Q1: Sex × time (full cohort, N = 179)

For each EEG measure $Y \in$ {frontal gamma power, auditory connectivity, speech connectivity, gamma lateralization}:

$$Y_{it} = \beta_0 + \beta_1\,\text{time}_t + \beta_2\,\text{sex}_i + \beta_3\,(\text{time} \times \text{sex})_{it} + \beta_4\,\text{site}_i + \beta_5\,\text{IQ}_i + u_i + \varepsilon_{it}$$

where $u_i \sim N(0, \sigma_u^2)$ is a by-subject random intercept.

Q1.2 Sex × ASD-likelihood × time (full cohort)

$$Y_{it} = \beta_0 + \beta_1\,\text{time} + \beta_2\,\text{sex} + \beta_3\,\text{group} + \beta_4\,(\text{time} \times \text{sex}) + \beta_5\,(\text{time} \times \text{group}) + \beta_6\,(\text{sex} \times \text{group}) + \beta_7\,(\text{time} \times \text{sex} \times \text{group}) + \beta_8\,\text{site} + \beta_9\,\text{IQ} + u_i + \varepsilon_{it}$$

where group ∈ {TLA (reference), ELA}.

Q1.3 Sex × ASD-diagnosis × time (ELA subsample only, N = 90; exploratory)

Same structure as Q1.2 but group is replaced by ASD-diagnosis ∈ {no-ASD (reference), ASD}.

Q2.1 Speech connectivity × sex × time on language growth

Speech connectivity was mean-centred across the full Q2 sample. Separate models were fitted for expressive and receptive language:

$$\text{Language}_{it} = \beta_0 + \beta_1\,\text{time} + \beta_2\,\text{speech\_con\_c} + \beta_3\,\text{sex} + \beta_4\,(\text{time} \times \text{speech\_con\_c}) + \beta_5\,(\text{time} \times \text{sex}) + \beta_6\,(\text{speech\_con\_c} \times \text{sex}) + \beta_7\,(\text{time} \times \text{speech\_con\_c} \times \text{sex}) + \beta_8\,\text{IQ} + \beta_9\,\text{site} + u_i + \varepsilon_{it}$$

Q3.1 / Q3.2 HC cluster × sex × time on language growth

Cluster assignment (A/B/C, derived without modification from Blanco-Gomez, Wright et al., 2025) was entered as a three-level factor. Separate models were fitted for expressive and receptive language:

$$\text{Language}_{it} = \beta_0 + \beta_1 \text{ time} + \beta_2 \text{ sex} + \beta_3 \text{ cluster} + \beta_4 \text{ (time} \times \text{sex)} + \beta_5 \text{ (time} \times \text{cluster)} + \beta_6 \text{ (sex} \times \text{cluster)} + \beta_7 \text{ (time} \times \text{sex} \times \text{cluster)} + \beta_8 \text{ site} + \beta_9 \text{ IQ} + u_i + \varepsilon_{it}$$

Holm correction family definitions

Each family contains all tests from **one research question** applied simultaneously across outcomes (or language measures).

**Family**	**Question**	**Tests included (k = 8 or 5 or 6)**
**Q1**	Do males and females differ in EEG development?	k = 8: sex-at-6-months × 4 EEG measures + sex×time × 4 measures
**Q1.2**	Does sex interact with ASD likelihood?	k = 8: sex×group × 4 measures + sex×group×time × 4 measures
**Q1.3** *(exploratory)*	Does sex interact with ASD diagnosis? (ELA only)	k = 8: sex×diagnosis × 4 measures + sex×diagnosis×time × 4 measures.
**Q2.1**	Is the speech connectivity–language relationship moderated by sex?	k = 5: expressive growth 3-way; receptive growth 3-way ; receptive 36-month interaction ; expressive sex×time; receptive sex×time
**Q3**	Do sex differences in language trajectories vary by HC cluster?	k = 6: sex×time, sex×cluster, sex×cluster×time

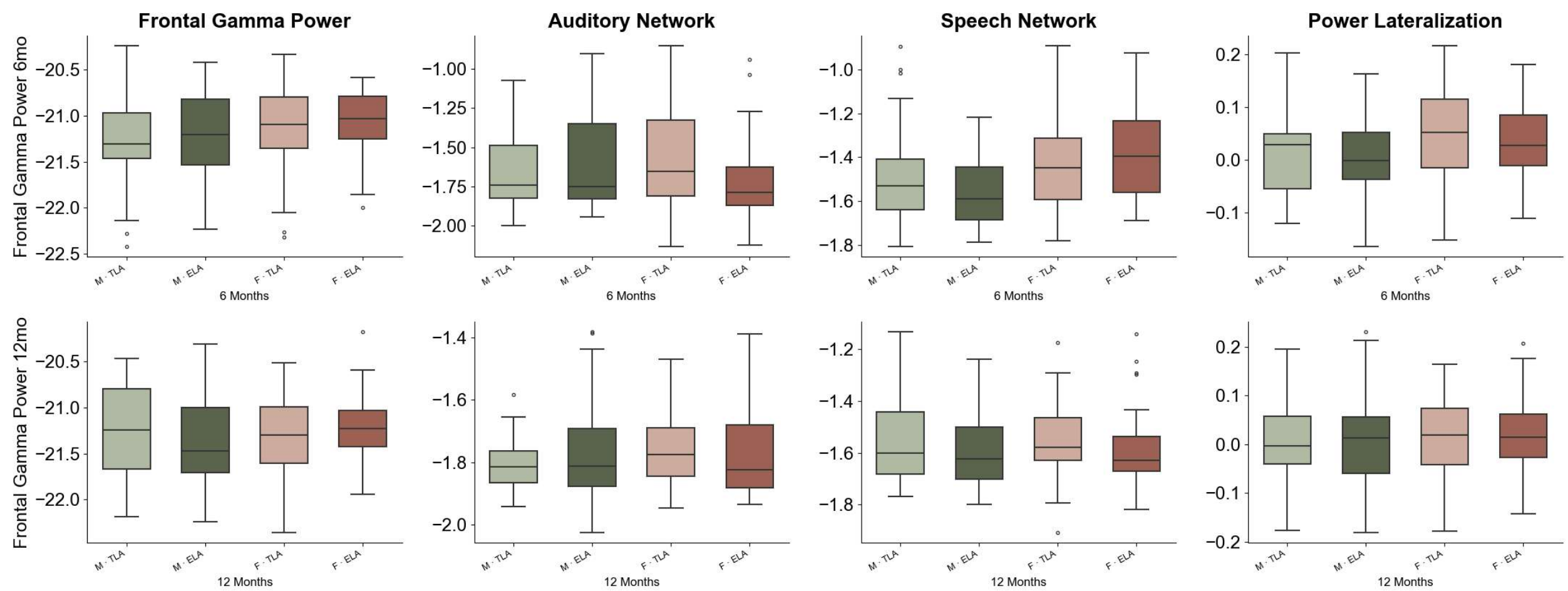

Supplementary Figure S1: Longitudinal trajectories of four frontal gamma power (log), auditory network connectivity (log), speech network connectivity (log), and power lateralization from 6 months (upper panels) to 12 months (lower panels). The dashed horizontal reference line in the Power Lateralization panel denotes zero (no hemispheric asymmetry

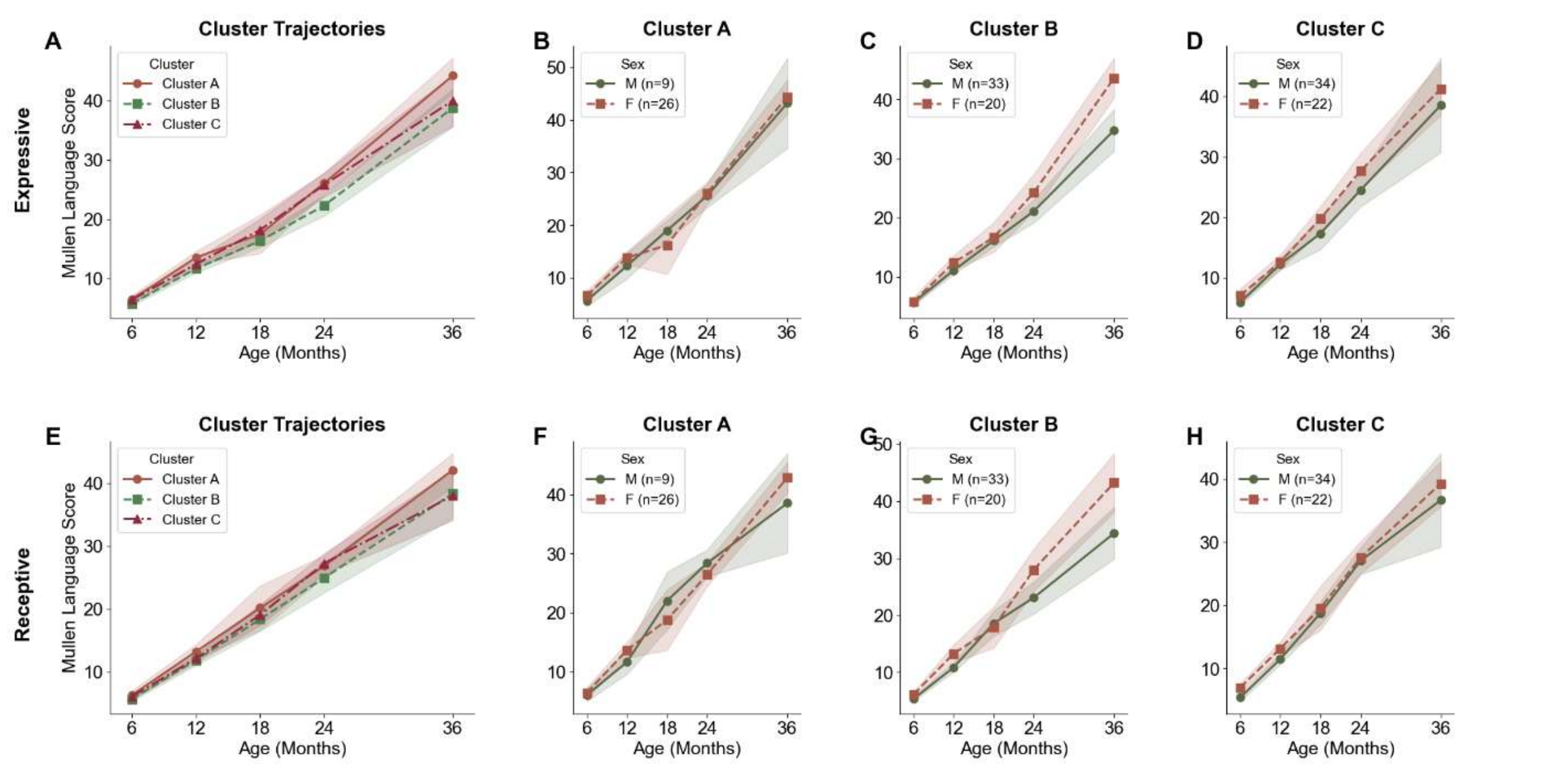

Supplementary Figure S3: **(A)** Expressive language trajectories for each class across all participants. **(B–D)** expressive language trajectories stratified by sex within Clusters A, B, and C, respectively. **(E)** Receptive language trajectories for each class across all participants. **(F-H)** Receptive language trajectories stratified by sex within Clusters A, B, and C, respectively.

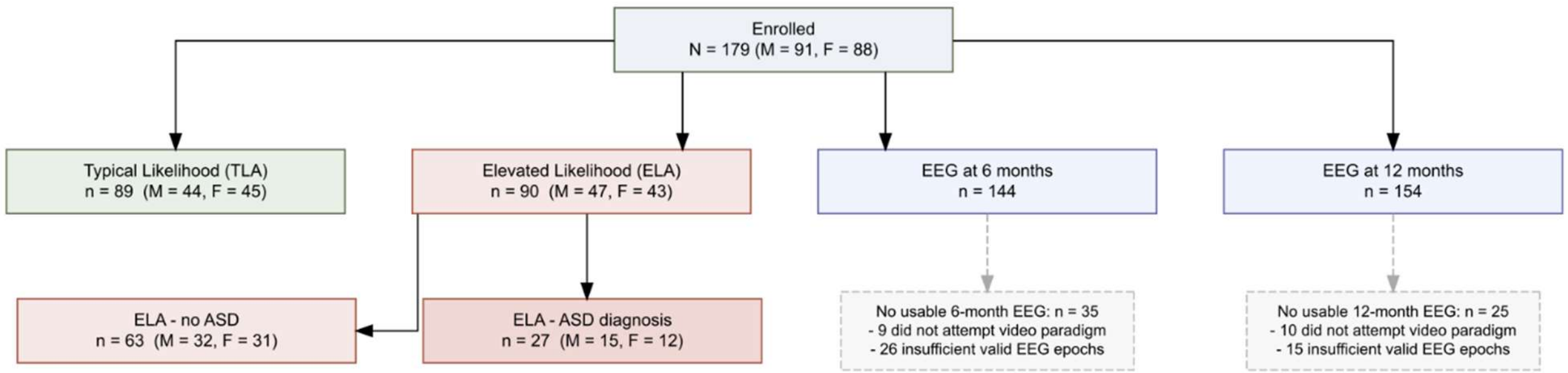

Supplementary Figure S4: Flow chart of participant enrolment.
